# Timing of Do-Not-Resuscitate Order Establishment Varies by Illness Trajectory and Clinical Context in Children Receiving Palliative Care: A Retrospective Cohort Study

**DOI:** 10.7759/cureus.115505

**Published:** 2026-08-31

**Authors:** Saba Javadi, Immaculada Espiritu, Tung Ming Leung, Oliver Karam, Linda B Siegel

**Affiliations:** 1 Pediatrics, Yale School of Medicine, New Haven, USA; 2 Pediatrics, Cohen Children’s Medical Center, New Hyde Park, USA; 3 Biostatistics, Northwell Health, New Hyde Park, USA

**Keywords:** advance care planning, do-not-resuscitate order, end-of-life decision-making, kaplan-meier survival analysis, life-sustaining therapy, pediatric critical care unit

## Abstract

Background

Do-not-resuscitate (DNR) orders in pediatrics are frequently analyzed as static outcomes, although they emerge through a longitudinal process shaped by evolving illness trajectories and repeated clinical encounters. Our primary objectives were to characterize the time from the first pediatric palliative care (PPC) consultation to formal DNR order establishment and to identify associated clinical factors. Secondary objectives were to describe longitudinal changes in DNR status and withdrawal of life-sustaining therapy.

Methodology

We conducted a retrospective cohort study of 371 children who received hospital-based PPC consultation at a quaternary children’s hospital in the United States between December 2011 and December 2016. The descriptive cohort included all 371 patients. Time-to-event analyses included 351 patients with an ascertainable event time and no DNR order before the first PPC consultation. Kaplan-Meier methods and multivariable Cox proportional hazards models were used, with follow-up extending across subsequent hospitalizations.

Results

Among 371 patients (median age = 2.4 years; 49.1% female), 115 (31.0%) had a documented DNR order. In the time-to-event cohort, 95 patients established a DNR order. DNR-free survival differed by diagnosis category (log-rank p = 0.003). Life-limiting illness without a realistic hope of cure was associated with a higher hazard of DNR establishment (adjusted hazard ratio (adjHR) = 2.10, 95% confidence interval (CI) = 1.14-3.86, p = 0.017). Symptom management consultations were associated with a lower hazard (adjHR = 0.08, 95% CI = 0.02-0.35, p < 0.001). Other hospital-based settings were associated with a higher hazard than the pediatric intensive care unit (adjHR = 5.85, 95% CI = 1.74-19.69, p = 0.004), although this estimate was based on six patients and three events. Religious affiliation was not associated with time to DNR. Among patients with a DNR order, 54 of 115 (47.0%) underwent withdrawal of life-sustaining therapy.

Conclusions

Formal DNR order establishment among children receiving palliative care was longitudinal and associated with illness trajectory and consultation context. DNR order establishment and withdrawal of life-sustaining therapy were distinct outcomes. Estimates from sparse subgroups require cautious interpretation.

## Introduction

With the advent of cardiopulmonary resuscitation (CPR) in the 1960s, physicians gained the ability to restore spontaneous circulation in patients who would otherwise have died from cardiac arrest. Although this innovation extended life in many circumstances, it also introduced the possibility of prolonging dying in the setting of irreversible disease. In response, hospitals began implementing do-not-resuscitate (DNR) orders in the 1970s to allow clinicians to withhold CPR when it was inconsistent with patient preferences or unlikely to provide meaningful benefit [1]. We use DNR throughout because it was the terminology recorded in the institutional electronic health record; in this study, it refers specifically to a formal order excluding CPR.

For adults with decision-making capacity, DNR orders can be established directly according to patient wishes. In pediatrics, however, decisions regarding resuscitation are typically made by surrogate decision-makers, most often parents or legal guardians, in collaboration with the treating physician and, when developmentally appropriate, with the assent of the child [2]. These decisions occur in complex emotional and ethical contexts, particularly when children have serious or life-limiting illnesses and families must weigh the potential burdens and benefits of continued life-sustaining treatment [3].

Prior literature in adult populations has demonstrated substantial variation in DNR status according to disease type, demographic characteristics, and geographic region [4,5]. In contrast, a more limited body of literature has examined the clinical and contextual factors associated with DNR order establishment in children. Existing pediatric studies suggest that prognosis, parental values, and cultural or religious considerations may influence decisions surrounding resuscitation [6-8]. However, much of this literature treats DNR status as a static attribute rather than a process that evolves over time.

In clinical practice, decisions regarding resuscitation are rarely made at a single discrete moment. Instead, they emerge through repeated discussions between clinicians and families as illness trajectories evolve, prognostic understanding develops, and goals of care are revisited. Parents often distinguish between withholding CPR and actively removing ongoing life-sustaining therapies [9-11]. This distinction is central to the present study because the primary outcome was formal DNR order establishment; withdrawal of other therapies was examined separately as a secondary descriptive outcome.

Pediatric palliative care (PPC) teams frequently facilitate communication regarding prognosis, goals of care, and treatment preferences throughout the disease trajectory. Many children receive palliative care for chronic, life-limiting conditions such as genetic syndromes and neurologic or neurodegenerative diseases, in which death may not be imminent [12,13]. Although DNR discussions represent only one component of pediatric advance care planning, and many children receiving PPC remain full code [12,13], the first PPC consultation provides a clinically meaningful and consistently documented landmark from which to examine subsequent formal DNR orders.

Previous studies have shown that PPC exposure facilitates earlier and more frequent advance care planning, including limitations on CPR [14-16]. Accordingly, DNR status may be better conceptualized as a dynamic clinical outcome rather than a fixed patient characteristic. Unlike the onset of illness or a change in prognosis, the first PPC consultation was consistently documented across the cohort and marked the beginning of specialty palliative care involvement. Our first objective was to characterize time from the first PPC consultation to formal DNR order establishment. Our second objective was to identify clinical factors associated with that timing. Secondary objectives were to describe changes in DNR status across hospitalizations and withdrawal of life-sustaining therapy. Because pediatric evidence was limited and the analysis was exploratory, no directional hypotheses were prespecified.

## Materials and methods

Study design and population

We conducted a retrospective cohort study of patients aged 0-21 years who received hospital-based consultation from a PPC team between December 2011 and December 2016 at a quaternary children’s hospital in the United States. This period was the interval covered by the original institutional review board-approved data collection. Institutional review board approval was obtained with a waiver of informed consent.

Patients were eligible if they had at least one documented hospital-based PPC consultation and ascertainable DNR status. Outpatient-only PPC contacts were outside the study scope. Analyses were conducted at the patient level, with one observation per patient. For patients with multiple hospitalizations, follow-up was anchored to the first PPC consultation and extended across subsequent admissions; admissions were not analyzed as separate clustered observations.

DNR status was ascertained from the formal electronic health record code-status order. A discussion without an order did not meet the outcome definition. A partial or limited code order was classified as DNR only when it explicitly excluded CPR. When a valid dated formal order conflicted with the binary DNR field, the dated formal order was used for classification. DNR status was selected because it was extractable as a discrete outcome, unlike the nuanced and often incompletely documented processes of family discussions, symptom relief, or family-reported quality of life.

Description of the pediatric palliative care team

Throughout the study period, the PPC team consisted of the same two attending physicians, both dual-trained in palliative medicine and pediatric critical care, together with a hospital chaplain. Staffing did not change during the study. The service cared for 371 unique patients across 556 consultation records, approximately 74 new patients per year. Consultations occurred at the discretion of the primary treating team, except that extracorporeal membrane oxygenation (ECMO) initiation triggered an automatic PPC consultation throughout the study period. Goals-of-care and DNR discussions were individualized; the primary treating team entered the formal DNR order.

Data collection

Data were abstracted by members of the PPC team who were manuscript coauthors using a standardized form. The form primarily captured objective fields, including dates, locations, formal orders, and disposition. Given the simplicity of these fields, each record underwent one extraction, and formal inter-rater reliability was not assessed. Abstractors relied on their clinical expertise for classification, and ambiguous diagnosis and consultation reason assignments were resolved by consensus between two authors.

Demographic data included age in years at the first PPC consultation, sex as documented in the medical record, and caregiver-reported primary language and religious affiliation. Other and unknown or deferred religious affiliations were retained as explicit categories rather than treated as missing. Clinical variables included primary diagnosis, dominant symptom at first consultation, location at first consultation, consultation reason, hospital length of stay before the first consultation, and duration of first PPC involvement. Location was categorized as the pediatric intensive care unit (PICU), neonatal intensive care unit (NICU), pediatric ward, or other hospital-based setting; the last category included the emergency department, psychiatry, and other inpatient settings.

Diagnosis categories were developed specifically for this study based on anticipated illness trajectory and were mutually exclusive: life-threatening illness with the possibility of cure; life-limiting illness with no realistic hope of cure; progressive condition for which treatment is palliative; and severe neurologic injury leading to susceptibility to health complications. When diagnoses overlapped, the condition judged to dominate the anticipated trajectory was assigned by consensus between two authors.

The reason for consultation was abstracted from the initial PPC note as goals-of-care or advance care planning, symptom management, automatic protocol-driven consultation at ECMO initiation, readmission follow-up, or other. Readmission follow-up referred to a subsequent hospitalization for a patient already known to the PPC service. Reasons were mutually exclusive; when more than one was documented, the dominant reason was assigned by consensus between two authors without a predefined hierarchy. For regression, the sparse automatic protocol, readmission follow-up, and other categories were combined. Patients with more than one symptom were assigned to the single symptom judged clinically dominant by the abstracting clinician. Other symptoms included pain, multisystem involvement, and feeding intolerance.

Withdrawal of life-sustaining therapy was defined as active removal of mechanical ventilation, vasoactive support, or ECMO. Withholding or non-escalation of treatment was not classified as withdrawal. Artificial nutrition was not included, and no patient in the cohort was receiving dialysis. Withdrawal status was recorded as undertaken, not undertaken, not applicable, brain death, or undocumented or indeterminate. Disposition was defined from the final recorded hospitalization as discharge alive or in-hospital death; the clinical unit was recorded as the site of death when applicable. Duration of first PPC involvement was calculated from the first PPC consultation to discharge from the hospitalization during which that consultation occurred. Overall PPC follow-up could span multiple hospitalizations. Length of stay and PPC duration were descriptive variables and were not used as predictors in the time-to-event model.

Primary outcome

The primary outcome was time in days from the first PPC consultation to the first formal DNR order. The order date, rather than the date of a discussion, was used as the event date. Orders entered on the date of the first consultation were assigned an event time of zero. Six patients with a formal DNR order before the first PPC consultation were included in descriptive analyses but excluded from time-to-event analyses because the outcome was present at baseline. A total of 14 additional patients with documented DNR status but no usable order date were retained descriptively but excluded from time-to-event analyses because their event time could not be established.

Time-to-event analysis

Time to DNR order was summarized using Kaplan-Meier methods. Time zero was the date of the first PPC consultation, and follow-up extended across subsequent hospitalizations. Interim discharge did not terminate follow-up. Patients without a DNR order were censored at the earliest of death, the last recorded hospital encounter, or the end of the study period. Curves were interpreted as the probability of remaining without a documented DNR order and not as cumulative incidence in the presence of death. Curves were stratified by diagnosis category and compared using the log-rank test. Confidence intervals (CIs) for median times were obtained by inversion of log-log Kaplan-Meier confidence bands.

Multivariable Cox proportional hazards regression estimated associations with the instantaneous rate of formal DNR order establishment among patients remaining DNR-free. Death was treated as a terminal censoring event, while discharge was not considered terminal because follow-up resumed if a patient returned. A Fine-Gray model was not used because the objective was association rather than population-level absolute incidence. Covariates were selected before modeling to represent illness trajectory, consultation context, care setting, and a potentially relevant family-level characteristic identified in prior literature: diagnosis category, consultation reason, location, and religious affiliation. Catholic affiliation was the reference category, and unknown or deferred affiliation was retained explicitly. Age, illness severity, duration of illness, and calendar year were not added post hoc because several were unavailable and the 95 events, together with sparse categories, limited additional model degrees of freedom. Adjusted hazard ratios (adjHRs), 95% CIs, and p-values were reported for all model terms. Tied event times were handled using the Breslow method. Proportional hazards assumptions were assessed graphically and using Schoenfeld residuals; no violations were detected, so stratification or time-varying coefficients were not required.

Secondary analyses

Among patients with a documented DNR order, withdrawal of life-sustaining therapy was summarized descriptively as a separate decision. Because the date of withdrawal was not consistently documented and withdrawal status was indeterminate in a substantial minority, time to withdrawal was not modeled.

Statistical analysis

Continuous variables were summarized using medians with interquartile ranges (IQRs). Categorical variables were summarized using frequencies and proportions. No model covariate was missing, and no imputation was performed. Predefined categories were retained, with sparse estimates interpreted cautiously. No baseline group comparison or post hoc subgroup tests were performed. Statistical significance was defined as a two-sided p-value below 0.05. All analyses were performed using SPSS Statistics for Windows, Version 31.0.1 (IBM Corp., Armonk, NY, USA).

## Results

Study population

During the study period, 378 pediatric patients had a recorded PPC consultation. Seven lacked ascertainable DNR status, yielding a descriptive cohort of 371 patients and 556 PPC consultation records. Median age at the first PPC consultation was 2.4 years (IQR = 0.1-13.3), and 182 of 371 (49.1%) patients were female. The caregivers’ primary language was English for 306 of 371 (82.5%) patients. The most commonly documented caregiver religious affiliations were Catholic (123 of 371, 33.2%), Christian (91 of 371, 24.5%), and Muslim (40 of 371, 10.8%). Other affiliations included Jewish (22 of 371, 5.9%) and Protestant (14 of 371, 3.8%), while 81 of 371 (21.8%) were recorded as other or unknown (Table 1).

**Table 1:** Demographic characteristics and hospitalization details of pediatric patients receiving palliative care consultation (N = 371). Age and hospital location refer to the time of the first palliative care consultation. Other settings include the emergency department, psychiatry, and other inpatient settings. Percentages use 371 as the denominator and may not sum to 100 because of rounding. IQR: interquartile range; PICU: pediatric intensive care unit; NICU: neonatal intensive care unit

Characteristic	Value
Age at first palliative care consultation, median (IQR), years	2.4 (0.1–13.3)
Female sex, n (%)	182 (49.1)
Primary caregiver language, n (%)	
English	306 (82.5)
Other language	65 (17.5)
Caregiver religious affiliation, n (%)	
Catholic	123 (33.2)
Christian	91 (24.5)
Muslim	40 (10.8)
Jewish	22 (5.9)
Protestant	14 (3.8)
Other or unknown	81 (21.8)
Hospital location at the first palliative care consultation, n (%)	
PICU	219 (59.0)
NICU	78 (21.0)
Pediatric ward	67 (18.1)
Other settings	7 (1.9)
Length of stay before the first consultation, median (IQR), days	6 (2.5–15.5)
Duration of the first palliative care involvement, median (IQR), days	14 (5–30)

At the first PPC consultation, 219 of 371 (59.0%) patients were in the PICU, 78 of 371 (21.0%) in the NICU, 67 of 371 (18.1%) in a pediatric ward, and 7 of 371 (1.9%) in other hospital-based settings, including the emergency department, psychiatry, and other inpatient settings. Median hospital stay before the first PPC consultation was six days (IQR = 2.5-15.5). Median duration of first PPC involvement, from the first consultation to discharge from that hospitalization, was 14 days (IQR = 5-30) (Table 1).

At the time of the first PPC consultation, primary diagnoses were categorized as life-threatening illness with the possibility of cure in 163 of 371 (43.9%) patients, life-limiting illness with no realistic hope of cure in 33 of 371 (8.9%), progressive condition for which treatment was palliative in 63 of 371 (17.0%), and severe neurologic injury leading to susceptibility to health complications in 112 of 371 (30.2%). The most common presenting symptom categories were respiratory in 121 of 371 (32.6%) and neurologic in 104 of 371 (28.0%), with 146 of 371 (39.4%) presenting with other symptoms, including pain, multisystem involvement, or feeding intolerance. A total of 87 of 371 (23.5%) patients experienced more than one hospitalization with PPC involvement during the study period (Table 2).

**Table 2:** Clinical characteristics at the time of the first palliative care consultation (N = 371). Each patient was assigned one clinically dominant symptom category. Other symptoms include pain, multisystem involvement, and feeding intolerance. Diagnosis categories are mutually exclusive and reflect anticipated illness trajectory. Percentages use 371 as the denominator and may not sum to 100 because of rounding.

Characteristic	N (%)
Primary diagnosis category	
Life-threatening illness with the possibility of cure	163 (43.9)
Life-limiting illness with no realistic hope of cure	33 (8.9)
Progressive condition for which treatment is palliative	63 (17.0)
Severe neurologic injury leading to susceptibility to complications	112 (30.2)
Presenting symptom category	
Respiratory symptoms	121 (32.6)
Neurologic symptoms	104 (28.0)
Other symptoms	146 (39.4)
Patients with more than one hospitalization involving palliative care	87 (23.5)

Do-not-resuscitate orders and withdrawal of life-sustaining therapy

In the descriptive cohort, 115 of 371 patients (31.0%) had a documented formal DNR order. Among the 87 patients with PPC involvement during more than one hospitalization, 25 (28.7%) had variation in documented DNR status across encounters. A total of 21 transitioned from no DNR to DNR at least once, while four had a DNR order documented during an earlier encounter but not during a later encounter (Table 3).

**Table 3:** Documented DNR orders, longitudinal status, withdrawal, and final disposition. Denominators are indicated in the row labels. The descriptive DNR denominator includes all 115 patients with a documented formal order, including those with indeterminate order timing. Patients with undocumented withdrawal status were retained in the denominator and were not classified as having no withdrawal. Disposition is based on the final recorded hospitalization. DNR: do-not-resuscitate; PPC: pediatric palliative care

Characteristic	N (%)
Patients with a documented DNR order (n = 371)	115 (31.0)
Variation in DNR status across hospitalizations (n = 87)	25 (28.7)
No DNR to DNR at least once	21 (24.1)
DNR documented earlier but not in a later encounter	4 (4.6)
Timing relative to the first PPC consultation (n = 115)	
Before consultation	6 (5.2)
On or after consultation	95 (82.6)
Timing indeterminate	14 (12.2)
Withdrawal of life-sustaining therapy (n = 115)	
Withdrawal undertaken	54 (47.0)
Withdrawal not undertaken	25 (21.7)
Not applicable	8 (7.0)
Brain death declared	2 (1.7)
Undocumented or indeterminate	26 (22.6)
Final disposition without a DNR order (n = 256)	
Discharged alive	225 (87.9)
In-hospital death	31 (12.1)
Final disposition with a DNR order (n = 115)	
Discharged alive	41 (35.7)
In-hospital death	74 (64.3)

Relative to the first PPC consultation, 6 of 115 patients (5.2%) had a formal DNR order beforehand, 95 (82.6%) had an order on or after the consultation, and timing could not be determined for 14 (12.2%).

Withdrawal of life-sustaining therapy was documented in 54 of 115 patients (47.0%) with a DNR order. Among patients without a DNR order, 225 of 256 (87.9%) were discharged alive, and 31 (12.1%) died in the hospital. Among patients with a DNR order, 41 of 115 (35.7%) were discharged alive, and 74 (64.3%) died in the hospital. Disposition refers to the final recorded hospitalization (Table 3).

Time from palliative care consultation to do-not-resuscitate order

The time-to-event cohort included 351 patients and 95 documented DNR events. Six patients with a DNR order before the first PPC consultation and 14 patients with documented DNR status but no usable order date were excluded from this analysis. With follow-up extending across hospitalizations, median time to first DNR order was 40 days (95% CI = 7-384) for patients with life-limiting illness without a realistic hope of cure, 388 days (95% CI = 286 to not estimable) for patients with progressive conditions for which treatment was palliative, and 479 days (95% CI = 228 to not estimable) for patients with severe neurologic injury. Among patients with life-threatening illness with the possibility of cure, median time was not reached; 29 of 161 (18.0%) established a DNR order. DNR-free survival differed across diagnosis categories (log-rank chi-square = 13.8, df = 3, p = 0.003) (Figure 1).

**Figure 1:**
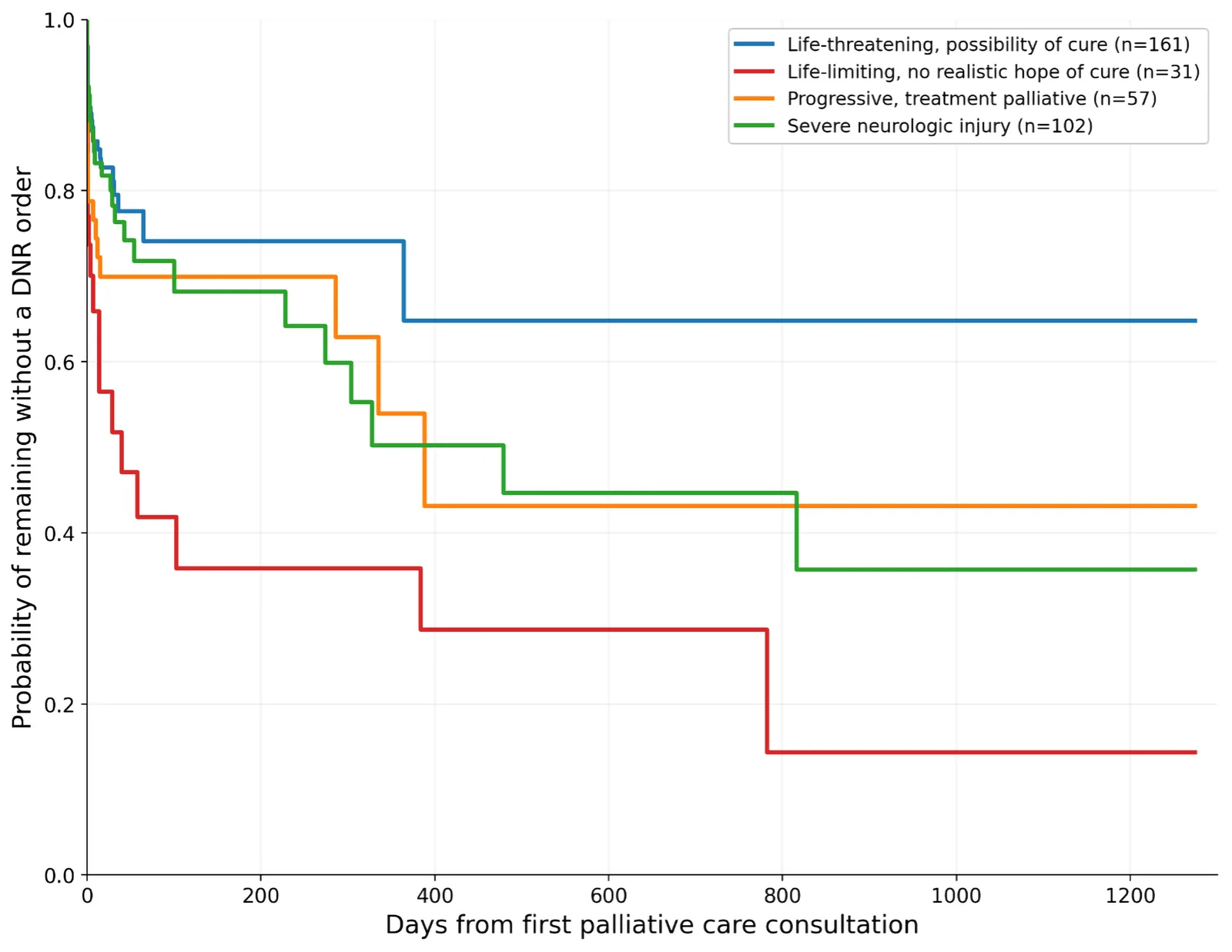
Kaplan-Meier curves for time from the first pediatric palliative care consultation to the first documented DNR order, stratified by diagnosis category. The vertical axis shows the probability of remaining without a documented DNR order and should not be interpreted as cumulative incidence in the presence of death. Time zero is the date of the first PPC consultation, and follow-up extends across subsequent hospitalizations. Patients without a DNR order were censored at the earliest of death, their last recorded hospital encounter, or the end of the study. Curves include life-threatening illness with the possibility of cure (n = 161), life-limiting illness without a realistic hope of cure (n = 31), progressive condition for which treatment was palliative (n = 57), and severe neurologic injury (n = 102). DNR: do-not-resuscitate

In the adjusted Cox model, consultation reason (overall p = 0.002) and location (overall p = 0.042) were associated with time to first DNR order. Symptom management consultations included 77 patients and two events and were associated with a lower hazard than goals-of-care consultations (adjHR = 0.08, 95% CI = 0.02-0.35, p < 0.001). The combined automatic protocol, readmission follow-up, and other category included 17 patients and three events and was not significantly different from goals-of-care consultations (adjHR = 0.43, 95% CI = 0.13-1.40, p = 0.162). Other hospital-based locations included six patients and three events and were associated with a higher hazard than the PICU (adjHR = 5.85, 95% CI = 1.74-19.69, p = 0.004); given the sparse data and wide CI, this estimate is hypothesis-generating.

Diagnosis category did not meet the threshold for an overall adjusted association (p = 0.058). Relative to life-threatening illness with the possibility of cure, life-limiting illness without a realistic hope of cure was associated with a higher hazard of DNR establishment (adjHR = 2.10, 95% CI = 1.14-3.86, p = 0.017), whereas progressive conditions (adjHR = 1.08, 95% CI = 0.57-2.02, p = 0.813) and severe neurologic injury (adjHR = 0.97, 95% CI = 0.57-1.65, p = 0.914) were not. Religious affiliation was not associated with time to DNR (overall p = 0.859). Proportional hazards testing identified no violation for any covariate group or globally (global p = 0.140) (Table 4).

**Table 4:** Multivariable Cox proportional hazards model of factors associated with time to first DNR order. The model included all 351 patients and 95 events, with no missing covariates. Hazard ratios are adjusted for diagnosis category, reason for PPC consultation, patient location, and religious affiliation. The No. (DNR events) column gives the number of patients and events in each category. Other settings include the emergency department, psychiatry, and other inpatient settings. Sparse estimates should be interpreted cautiously. CI: confidence interval; DNR: do-not-resuscitate; PICU: pediatric intensive care unit; NICU: neonatal intensive care unit

Variable	No. (DNR events)	Adjusted hazard ratio (95% CI)	P-value
Diagnosis category			0.058
Life-threatening illness with possibility of cure	161 (29)	Reference	-
Life-limiting illness without a realistic hope of cure	31 (18)	2.10 (1.14–3.86)	0.017
Progressive condition with palliative treatment	57 (19)	1.08 (0.57–2.02)	0.813
Severe neurologic injury	102 (29)	0.97 (0.57–1.65)	0.914
Reason for PPC consultation			0.002
Goals-of-care consultation	257 (90)	Reference	-
Symptom-management consultation	77 (2)	0.08 (0.02–0.35)	<0.001
Automatic protocol, readmission follow-up, or other	17 (3)	0.43 (0.13–1.40)	0.162
Patient location at consultation			0.042
PICU	208 (65)	Reference	-
NICU	70 (17)	1.07 (0.61–1.88)	0.820
Pediatric ward	67 (10)	1.20 (0.56–2.55)	0.637
Other hospital-based setting	6 (3)	5.85 (1.74–19.69)	0.004
Caregiver religious affiliation			0.859
Catholic	113 (31)	Reference	-
Christian	86 (27)	1.03 (0.61–1.76)	0.900
Jewish	22 (4)	0.92 (0.32–2.68)	0.885
Muslim	39 (13)	0.78 (0.40–1.53)	0.466
Unknown or deferred	18 (4)	0.95 (0.32–2.79)	0.920
Other	59 (13)	0.70 (0.36–1.35)	0.286
Protestant	14 (3)	0.55 (0.17–1.84)	0.334

Withdrawal of life-sustaining therapy among patients with do-not-resuscitate orders

Among the 115 patients with a documented DNR order, withdrawal of life-sustaining therapy was documented in 54 (47.0%) and explicitly not undertaken in 25 (21.7%). Withdrawal was recorded as not applicable in eight (7.0%) patients, two (1.7%) were declared brain dead, and withdrawal status was undocumented or indeterminate in 26 (22.6%). These patients were retained in the denominator rather than counted as having no withdrawal. Because the date of withdrawal was not consistently documented, withdrawal was not modeled as a time-to-event outcome.

## Discussion

In this retrospective cohort of children receiving specialty palliative care consultation, a formal DNR order was documented in approximately one-third of patients, with substantial variation in timing across diagnostic trajectories and consultation contexts. Modeling DNR status longitudinally emphasizes that a formal order may emerge after repeated clinical encounters rather than being a fixed patient attribute. These findings are associative and do not establish that PPC consultation or any measured characteristic caused earlier or later DNR establishment.

Illness trajectory was associated with DNR timing in a specific comparison but did not uniformly determine it. Children with life-limiting illness without a realistic hope of cure had a higher adjusted hazard of DNR establishment than those with potentially curable life-threatening illness, consistent with prior literature on illness trajectory and decisions about life-sustaining treatment [6,17]. However, the overall diagnosis category test narrowly exceeded the conventional significance threshold, and timing varied widely within categories. Prognostic category alone therefore does not explain when families and clinicians establish a DNR order.

The documented reason for consultation was associated with DNR timing. Goals-of-care consultations were followed by earlier DNR establishment than consultations focused primarily on symptom management. Because consultation reason was abstracted from the referral context, this association likely reflects differences in the clinical circumstances prompting consultation rather than an effect of PPC itself [18]. As only two DNR events occurred in the symptom management group, the magnitude of that estimate should be interpreted cautiously. The higher hazard estimated for other hospital-based settings was likewise based on only six patients and three events and requires external validation.

Importantly, DNR order establishment and withdrawal of life-sustaining therapy were distinct outcomes. Just under half of patients with a DNR order also underwent active removal of mechanical ventilation, vasoactive support, or ECMO. Prior studies suggest that parents often distinguish ethically and emotionally between withholding CPR and discontinuing ongoing therapies [9-11]. Our findings reinforce that these decisions may occur separately and are influenced by evolving clinical circumstances rather than solely by code status.

We did not observe an adjusted association between caregiver-reported religious affiliation and DNR timing. Recorded affiliation is an incomplete proxy for personal beliefs or the role of faith in a particular decision, and the finding should not be interpreted as evidence that religious considerations are unimportant [7,14]. Among the 87 patients with PPC involvement during multiple hospitalizations, 25 had variation in documented DNR status; most changed from no DNR to DNR, while four had a DNR order during an earlier encounter but not a later one. This bidirectional variation supports the concept that code status may evolve longitudinally.

PPC practice has evolved substantially since the study period. Contemporary programs increasingly emphasize longitudinal advance care planning and repeated goals-of-care discussions throughout the disease trajectory [15,19]. Although formal DNR orders represent only one component of advance care planning, they remain an important documented marker of evolving treatment preferences and prognostic understanding. The present findings suggest that DNR timing is associated with illness trajectory and consultation context, while leaving many family-, clinician-, and system-level influences unmeasured.

Together, these findings support longitudinal PPC involvement and iterative goals-of-care discussions as illness trajectories evolve. They also underscore the importance of distinguishing a formal DNR order from broader treatment limitation preferences when communicating with families and studying pediatric end-of-life care.

Limitations

Several limitations should be considered. This retrospective, single-center cohort included only children referred to PPC, and most referrals occurred at the discretion of the primary treating team. Referral and selection bias may therefore limit generalizability to children who were not referred, community hospitals, or programs with different referral models. As follow-up was limited to one institution, DNR orders, PPC encounters, or hospitalizations elsewhere were not captured. The study relied on formal orders and may not reflect the timing or content of family discussions. Because death was censored, Kaplan-Meier curves describe remaining DNR-free and should not be interpreted as population-level cumulative incidence.

Retrospective assignment of study-specific diagnosis, consultation reason, and dominant symptom categories may have introduced misclassification despite consensus review for ambiguous cases. A total of 14 patients with documented DNR status but no usable order date were excluded from time-to-event analyses, and withdrawal status was undocumented or indeterminate for 26 patients; bias is possible if unavailable documentation was not random. Estimates for other hospital locations and symptom management consultations were based on few events and may be unstable. Caregiver-reported religious affiliation does not measure personal beliefs, religiosity, or the role of faith in decision-making. Family structure, employment, financial circumstances, social support, illness severity, clinician communication, and prognostic uncertainty were not systematically captured and may contribute to residual confounding. Although PPC staffing was stable, referral patterns, documentation, and institutional practice may have changed from 2011 to 2016. Calendar year was not added to the model because the limited number of events and sparse categories increased the risk of overfitting. Prospective multicenter studies with standardized documentation are needed.

## Conclusions

Among children receiving specialty PPC, formal DNR order establishment occurred longitudinally and varied across illness trajectories and consultation contexts. Life-limiting illness without a realistic hope of cure was associated with a higher hazard of order establishment in a specific adjusted comparison, while symptom management consultations were associated with a lower hazard. Caregiver-reported religious affiliation was not independently associated with timing. These associations do not establish causation, and estimates based on sparse subgroups require cautious interpretation. A formal DNR order and withdrawal of life-sustaining therapy were distinct clinical outcomes, with fewer than half of patients with a DNR order also undergoing withdrawal. Clinicians should not treat code status as a proxy for broader treatment limitation preferences. Prospective multicenter studies that capture family, clinician, socioeconomic, and healthcare system factors and follow patients across care settings are needed to clarify the trajectory of pediatric advance care planning.
